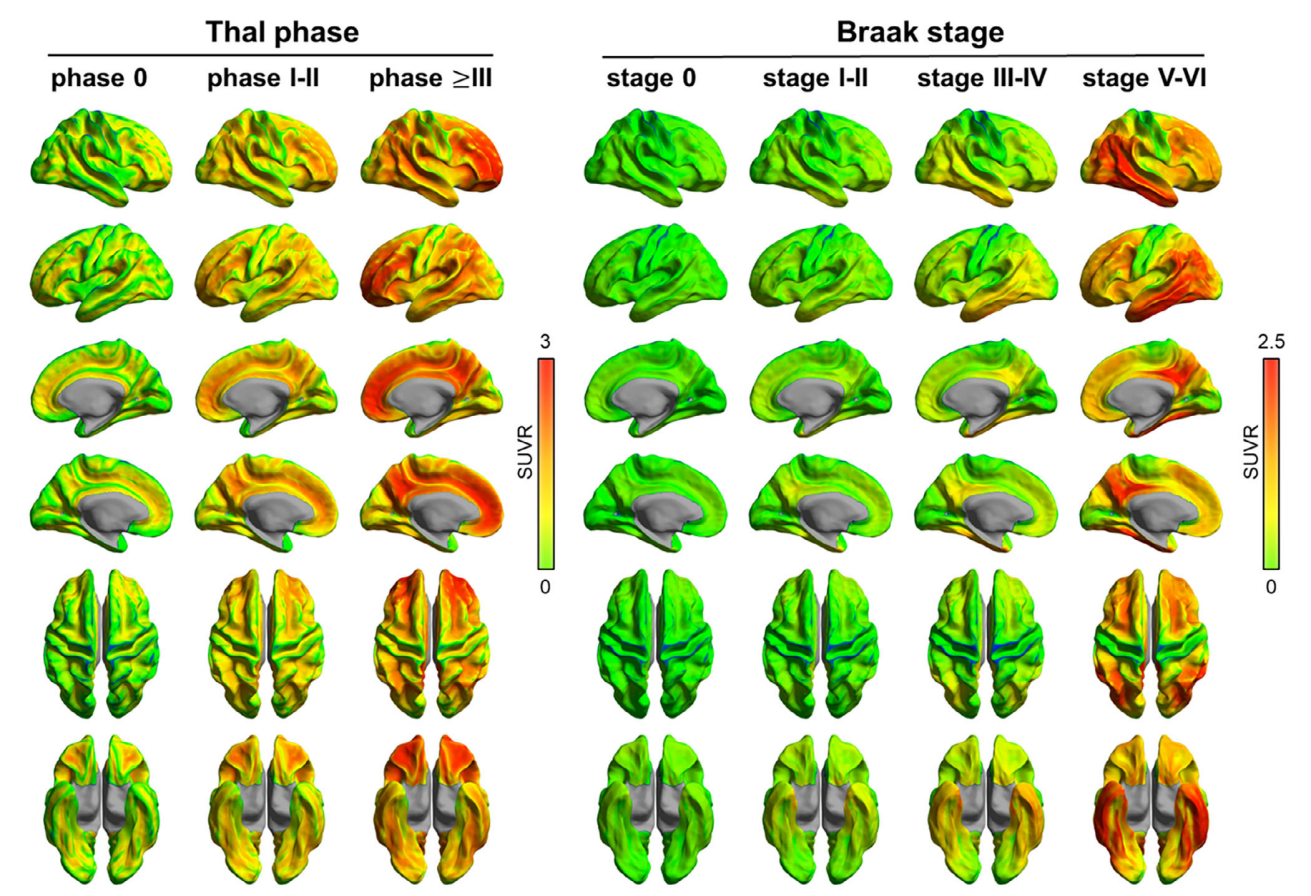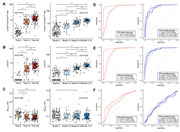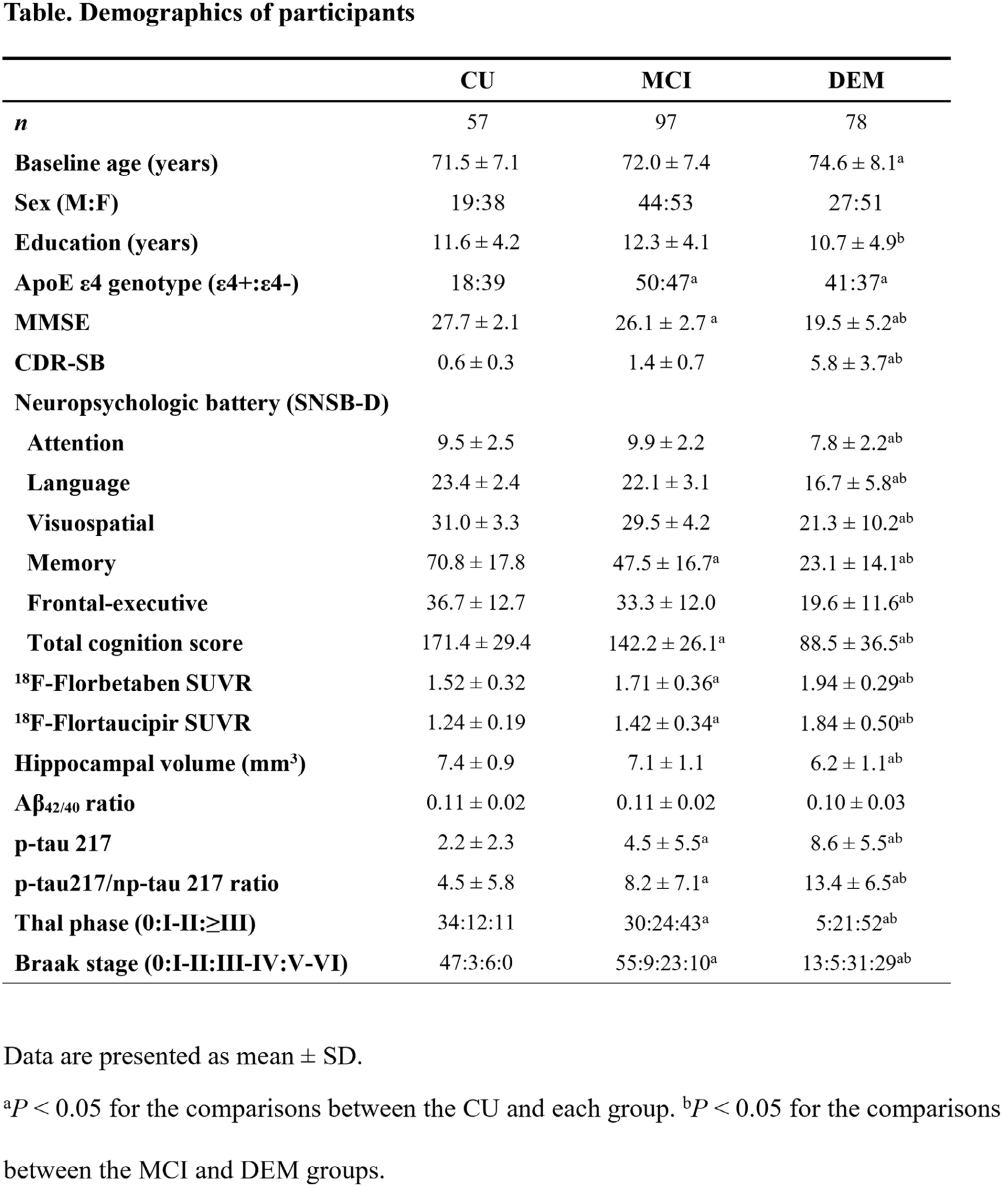# Predicting PET‐Based Amyloid and Tau Pathology Stages With Plasma Biomarkers in Alzheimer's disease

**DOI:** 10.1002/alz.090581

**Published:** 2025-01-09

**Authors:** Han‐Kyeol Kim, Jae Hoon Lee, Joong‐Hyun Chun, Jeong‐Ha Lee, Tim West, Kristopher M. Kirmess, Philip B. Verghese, Joel B. Braunstein, Daniel Connell, Young Hoon Ryu, Chul Hyoung Lyoo, Hanna Cho

**Affiliations:** ^1^ Wonju Severance Christian Hospital, Yonsei University Wonju College of Medicine, Wonju Korea, Republic of (South); ^2^ Gangnam Severance Hospital, Yonsei University College of Medicine, Seoul Korea, Republic of (South); ^3^ Severance Hospital, Yonsei University College of Medicine, Seoul Korea, Republic of (South); ^4^ C2N Diagnostics, LLC, Saint Louis, MO USA

## Abstract

**Background:**

We aimed to investigate whether the quantitative analysis of plasma biomarkers could distinguish the pathology stages indicated by positron emission tomography (PET)‐based Thal phase of amyloid and Braak stage of tau.

**Method:**

A total of 232 participants were enrolled, all of whom underwent ^18^F‐florbetaben (FBB), ^18^F‐flortaucipir (FTP) PET, plasma p‐tau217/np‐tau217 ratio, p‐tau217, and Aβ_42/40_ ratio. To differentiate between image‐based Thal phases and Braak stages, region‐of‐interests (ROIs) were constructed, and cut‐off points were established at each stage using Gaussian mixture modeling. Consequently, FBB PET was classified into Thal phase 0, I‐II, and ≥III groups, while FTP PET was categorized into Braak stage 0, I‐II, III‐IV, and V‐VI.

**Result:**

The plasma biomarker that most accurately reflected both the PET‐based Thal phase and the PET‐based Braak stage was the p‐tau217/np‐tau217 ratio. P‐tau 217 was superior in reflecting the Braak stage, but inferior to the p‐tau217/np‐tau217 ratio in the Thal phase. The Aβ_42/40_ ratio exhibited a difference between Thal phase 0 and I‐II, ≥III, but failed to distinguish between I‐II and ≥III, and did not correlate with the Braak stage. The AUC for predicting Thal phase I‐II and Thal phase ≥III using the p‐tau217/np‐tau217 ratio was 0.965 and 0.848, respectively. The AUC for predicting Braak I‐II, Braak III‐IV, and Braak V‐VI using the p‐tau217/np‐tau217 ratio was 0.864, 0.925, and 0.889, respectively.

**Conclusion:**

The p‐tau217/np‐tau217 ratio demonstrates a strong correlation with the pathological stages based on amyloid and tau PET and exhibits the ability to predict the pathological stages of Alzheimer's disease through quantitative values, particularly reflecting the earlier stages more accurately.